# Forsythiaside inhibits bacterial adhesion on titanium alloy and attenuates Ti-induced activation of nuclear factor-κB signaling-mediated macrophage inflammation

**DOI:** 10.1186/s13018-018-0834-x

**Published:** 2018-06-05

**Authors:** Haifeng Li, Dongmei Tang, Chao Qi, Xia Zhao, Guangchao Wang, Yi Zhang, Tengbo Yu

**Affiliations:** 1 Department of Sports Medicine, The Affiliated Hospital of Qingdao University, No. 16 Jiangsu Road, Qingdao, 266003 China; 2 Department of Anesthesiology, The Affiliated Hospital of Qingdao University, Qingdao, 266003 China; 3 Department of Orthopedics, Changhai Hospital, the Second Military Medical University, Shanghai, China

**Keywords:** Titanium, Forsythiaside, Anti-bacteria, Anti-inflammation, Nuclear factor-κB signaling pathway

## Abstract

**Background:**

Inflammation and biofilm formation by *Staphylococcus aureus* (*S. aureus*) are common causes of periprosthetic infection and loosening. Recently, we identified that forsythiaside is bacteriostatic for *S. aureus* and methicillin-resistant *S. aureus* (MRSA). The purpose of the present study was to examine the effect of forsythiaside on *S. aureus* and MRSA adhesion and biofilm formation on the surface of titanium alloy, which is a popular material for orthopedic joint prostheses.

**Methods:**

Two strains of *S. aureus* and MRSA were used for in vitro experiments. The spread plate method, confocal laser scanning microscopy (CLSM), and scanning electron microscopy (SEM) were used to characterize antimicrobial activity of forsythiaside. Real-time polymerase chain reaction (RT-PCR) and western blotting were used to investigate the inhibitory level of forsythiaside required for titanium-associated inflammation.

**Results:**

Direct colony counting showed that 16 μg/mL forsythiaside significantly inhibited *S. aureus* and MRSA adhesion on titanium alloy discs in 2 h. CLSM and SEM showed that higher concentrations (> 30 mg/mL) of forsythiaside effectively inhibited the adhesion of *S. aureus* and MRSA on the surface of the titanium disc in 24 h. Forsythiaside was capable of attenuating Ti-induced activation of nuclear factor-κB signaling, targeting IκB kinase-α (IKKα) kinases of macrophages, and influencing the expression of NF-κB downstream cytokines.

**Conclusions:**

These observations suggest that forsythiaside is a potential agent for the treatment of Ti implant-associated infection and inflammation.

## Background

Titanium (Ti) and its alloys are widely used in orthopedic implants such as hip and knee prostheses, fixations, and dental implants. The main disadvantages of using orthopedic implants are the chances of developing aseptic loosening and infection [1]. Once implanted, orthopedic prostheses may develop microbial infections, especially those used in open fracture fixation and arthroplasty [2, 3]. Two main reasons may contribute to Ti implant-associated infections. One is that Ti is bio-inert and an easy substratum for bacterial surface adhesion and biofilm formation. Therefore, Ti implant-associated infections can be inhibited by immobilizing antibacterial agents on the Ti surface. The other reason is that the host factors required for implantation of the Ti prosthesis belong to a relatively immune fade zone and a small bacterial load can lead to the severe Ti implant-associated infection [4]. Since Ti surfaces are susceptible to bacterial adherence and biofilm formation, surface modification of Ti is an important approach for fabricating antibacterial Ti implants. In the past decade, efforts on biofunctionalization of Ti were mainly focused on immobilization of antibacterial agents on the Ti surface; Ti surface-immobilized antibiotics, antimicrobial peptide (AMP), or inorganic ions are either bactericidal on contact or locally release antibacterial agents. However, cytotoxicity and local burst release of the antibacterial agents and bacterial resistance are common problems associated with such biofunctional Ti surfaces [5].

Forsythiaside has been widely used in traditional medicines in Asia to treat gonorrhea, erysipelas, inflammation, pyrexia, ulcer, and other diseases. Forsythiaside possesses strong antioxidative, antibacterial, anti-inflammatory, and cyclic adenosine activity [6, 7] and monophosphate phosphodiesterase (cAMP) inhibitory effects. Further, forsythiaside exhibited anti-oxidative abilities, since it can counter the adverse effects of endotoxins by decreasing the percentage of regulatory T cells (Tregs) and inhibiting the TLR4/MyD88/NF-κB signaling pathway [8]. In addition, forsythin also triggered weight loss and inhibited cigarette smoke-induced NF-κB activation in a dose-dependent manner with upregulation of Nrf2 and HO-1 expression [9]. Therefore, forsythiaside can be used as an alternative antioxidative and antibacterial agent of natural origin. In this study, we investigated the antibacterial activity of forsythiaside on bacterial adhesion on Ti. The anti-inflammatory role of THP-1-deprived macrophages toward Ti was also examined.

## Methods

The following reagents were used in this study: forsythiaside (Solarbio, China), Ti6AL4V (Titanium alloy, NaOH, Tris–HCl buffer (pH = 8.6), cDNA synthesis kit, real-time PCR kit (SYBR Premix EX Taq, TaKaRa), Pierce™ bicinchoninic acid (BCA) protein assay kit (ThermoFisher), RNA mini kit (Qiagen), alpha minimum essential medium (α-MEM, Hyclone), fetal bovine serum (FBS, Gibco, Australia), trypsin–EDTA (0.5%), Alexa Fluor 488 secondary antibody, IMMULITE®/IMMULITE®1000TNF-α and IMMULITE®/IMMULITE®1000 IL-1β, (SIEMENS), and the NF-κB signaling pathway kit (Cell Signaling Technology, USA).

### Antibacterial activity assay of forsythiaside

The minimum inhibitory concentration (MIC) was evaluated by the broth microdilution method recommended by the National Committee for Clinical Laboratory Standards (NCCLS) using the Mueller–Hinton broth (MHB) medium. In brief, the bacterial cell counts were adjusted to approximately 2 × 10^6^ colony-forming units (CFU)/mL, and the forsythiaside solution was diluted to 2048, 1024, 512, 256, 128, 64, 32, 16, and 8 μg/mL. A 100-μL volume of each bacterial suspension was added to the wells of a sterile 96-well plate containing 100 μL of each concentration of forsythiaside; the final volume in each well was 200 μL. Controls were prepared using the culture medium. The MIC was defined as the minimum forsythiaside concentration that inhibited bacterial growth.

### Bacterial adhesion on Ti

The spread plate method was used to investigate the effect of forsythiaside on bacterial adhesion on titanium [10]. Briefly, bacteria were diluted to 1.0 × 10^6^ CFU/mL with fresh tryptic soya broth (TSB) medium containing 10 or 50 μM forsythiaside and added to a standard flat-bottomed 96-well culture plate such that the final volume of the solution in the well was 200 μL. The culture was incubated statically at 37 °C for 24 h, after which the broth was carefully decanted and the adhered cells were washed thrice gently with 0.01 M phosphate-buffered saline (PBS) (pH 7.4) to eliminate free planktonic bacteria. Following ultrasonic cleaning of the Ti surface, serial dilutions of the bacterial culture were made and the spread plate method was used to quantitatively characterize the number of viable bacteria adhered to the flat Ti surface in the presence of different concentrations of forsythiaside at 6 and 24 h.

### Bacterial morphology

After disinfection at high temperature and pressure, the titanium plates (1 mm × 5 mm) were placed in 24-well plates and 1 mL fresh TSB bacterial suspension (*S. aureus* and MRSA), diluted to 1.0 × 10^6^ CFU/mL with fresh TSB medium containing 10 or 50 μM forsythiaside, was added in each well of each group. The cultures were incubated at 37 °C for 24 h, after which they were gently removed from the titanium plates and washed thrice with PBS to eliminate non-adherent planktonic bacteria. The Live/Dead Baclight™ viability kit was used to enumerate viable and dead bacteria. Briefly, the Ti substrates were stained for 15 min at room temperature per manufacturer’s instructions, followed by aspiration of the dye solution and gentle washing with PBS to remove non-specific staining. Live or dead bacteria were observed under a laser confocal microscope (Leica TCS, SP2, Germany). The live bacteria fluoresced green in the presence of the fluorescent dye SYSTO9, whereas the dead bacteria fluoresced red in the presence of PI.

For characterizing bacterial morphology by SEM, each group was removed from the small titanium plate after 24 h of culture and gently rinsed thrice with PBS to eliminate non-adherent floating bacteria. Glutaraldehyde solution (2.5%) was used for initial fixation at 4 °C for 2 h, followed by rinsing thrice with PBS for 1 h. Cover glass slides were fixed in 0.1% osmium tetroxide solution for 1 h. The cells were dehydrated using an alcohol gradient (30, 50, 70, 80, 90, 95, and 100%; 10 min for each concentration) and sprayed with gold at critical point dry time. The bacterial morphology and numbers were observed under a scanning electron microscope (SEM, JEOL JSM-6360LV, Japan).

### Macrophage viability on Ti

The human macrophage cell line THP-1 (Chinese Academy of Sciences, Shanghai, China) was cultured in Roswell Park memorial Institute (RPMI) 1640 medium supplemented with 10% FBS at 37 °C in a humidified 5% CO_2_ incubator. The macrophages were induced with phorbol-12-myristate-13-acetate (PMA) at the concentration of 100 ng/mL for 24 h. The Ti substrates were plated in 24-well plates prior to seeding of cells at the density of 5 × 10^5^/mL. After incubation with Ti substrates containing 0, 10, and 50 μM forsythiaside for 24 h, macrophage cell viability on each substrate was determined using the CCK-8 kit.

### Real-time PCR

The Ti substrate size used for RNA extraction was 34 mm × 2 mm. THP-1-deprived macrophages were seeded at 5 × 10^4^ cells per Ti discs and cultured in six-well plates containing the Ti substrates. Low concentration (10 μM) and high concentration (100 μM) of forsythiaside were added to the plates and incubated for 24 h. Total RNA was isolated from each Ti group using the RNA Mini kit after 3 days of co-culture. Five hundred nanograms RNA from each Ti sample was reversed-transcribed using the PrimeScript™ reverse transcription kit. The expression of the housekeeping gene glyceraldehyde-3-phosphate dehydrogenase (GAPDH) was used as an internal control to normalize the results obtained using the 2-ΔΔCT method. The sequences of primers used for real-time PCR analysis are shown in Table 1. Nos2 (iNOS and CCR-7) was selected as a marker for the M1 inflammatory phenotype, and CD206 and CD163 were selected as markers for the M2 regenerative phenotype [11]. Further, after 3 days of incubation of the Ti substrates with THP-1-deprived macrophages and different concentrations of forsythiaside, the levels of the pro-inflammatory cytokines (TNF-α, IL-1β, IL-6, and IL-8) in the cell supernatant was determined using the IMMULITE/IMMULITE 1000 TNF-α, IL-1β, and IL-8 kits, respectively (Siemens Healthcare Diagnostics Inc.), according to the manufacturer’s instructions.

**Table 1:** MIC of forsythiaside toward *S. aureus*, methicillin-resistant *S. aureus* (MRSA), *Staphylococcus epidermidis*, and methicillin-resistant *S. epidermidis* (MRSE)

μg/mL	256	128	64	32	16	8	4	Blank
*S. aureus* (ATCC 25923)	–	–	–	+	+	+	+	+
*S. epidermidis* (ATCC 35984)	–	–	–	+	+	+	+	+
MRSA (ATCC 43300)	–	–	+	+	+	+	+	+
MRSE	–	–	+	+	+	+	+	+

+ muddy, − clear

### Immunofluorescence

After 3 days of incubation of the Ti substrates with THP-1-deprived macrophages in the presence of different concentrations of forsythiaside, the Ti substrates were washed with PBS to detach the non-adherent cells and fixed with 4% paraformaldehyde overnight. Then incubated with 0.1% Triton X-100 for 15 min, gently washed with PBS, the cytoskeleton was stained with fluorescent phalloidin for 30 min according to the manufacturer’s protocol, and the macrophage cell morphology was visualized by fluorescence microscope (Nikon, Japan).

### Western blotting

After 2 and 4 h of incubation of Ti substrates with THP-1-deprived macrophages in the presence of different concentrations of forsythiaside, total proteins were extracted from cultured cells using radioimmunoprecipitation assay (RIPA) lysis buffer containing 0.1% phenylmethane sulfonyl fluoride (PMSF). Lysates were centrifuged at 15,000×*g* for 15 min, and the supernatants were collected. Protein concentration was determined using the BCA assay. Then, each protein lysate was resolved using sodium dodecyl sulfate–polyacrylamide gel electrophoresis (SDS–PAGE) on 10% gels and transferred to polyvinylidene difluoride membranes (Millipore, USA). The membranes were blocked with 5% skimmed milk in Tris-buffered saline–Tween 20 (TBST) solution for 1 h and then incubated with primary antibodies IKK-α, phosphorylated IKK-α, IκB-α, phosphorylated IκB-α, MyD88, and GAPDH (Cell Signaling Technology, USA) diluted in 1% (*w*/*v*) skimmed milk powder in TBST overnight at 4 °C. Membranes were washed and incubated with the appropriate secondary antibodies. Antibody reactivity was detected by exposure in the Taton imaging system.

### Statistical analysis

Data were expressed as mean ± standard error of mean (SEM), and experiments were performed in triplicate. The SPSS (version 19.0) was used to analyze the data. One-way ANOVA is followed by an S-N-K test to evaluate the differences between groups. *p* values < 0.05 were considered statistically significant.

## Results

### MIC of forsythiaside

The antimicrobial activity of forsythiaside was tested against four Gram-positive bacteria that are the common causes of orthopedic implant-related infections, namely, *S. aureus* (ATCC 25923), methicillin- resistant *S. aureus* (MRSA, ATCC 43300), *Staphylococcus epidermidis* (ATCC 35984), and methicillin-resistant *S. epidermidis* (MRSE). The MIC was 64 μg/mL for *S. aureus* and *S. epidermidis* and 128 μg/mL for MRSA and MRSE (Table 1).

### Characterization of the viable bacteria on Ti samples in the presence of forsythiaside

**Fig. 1:**
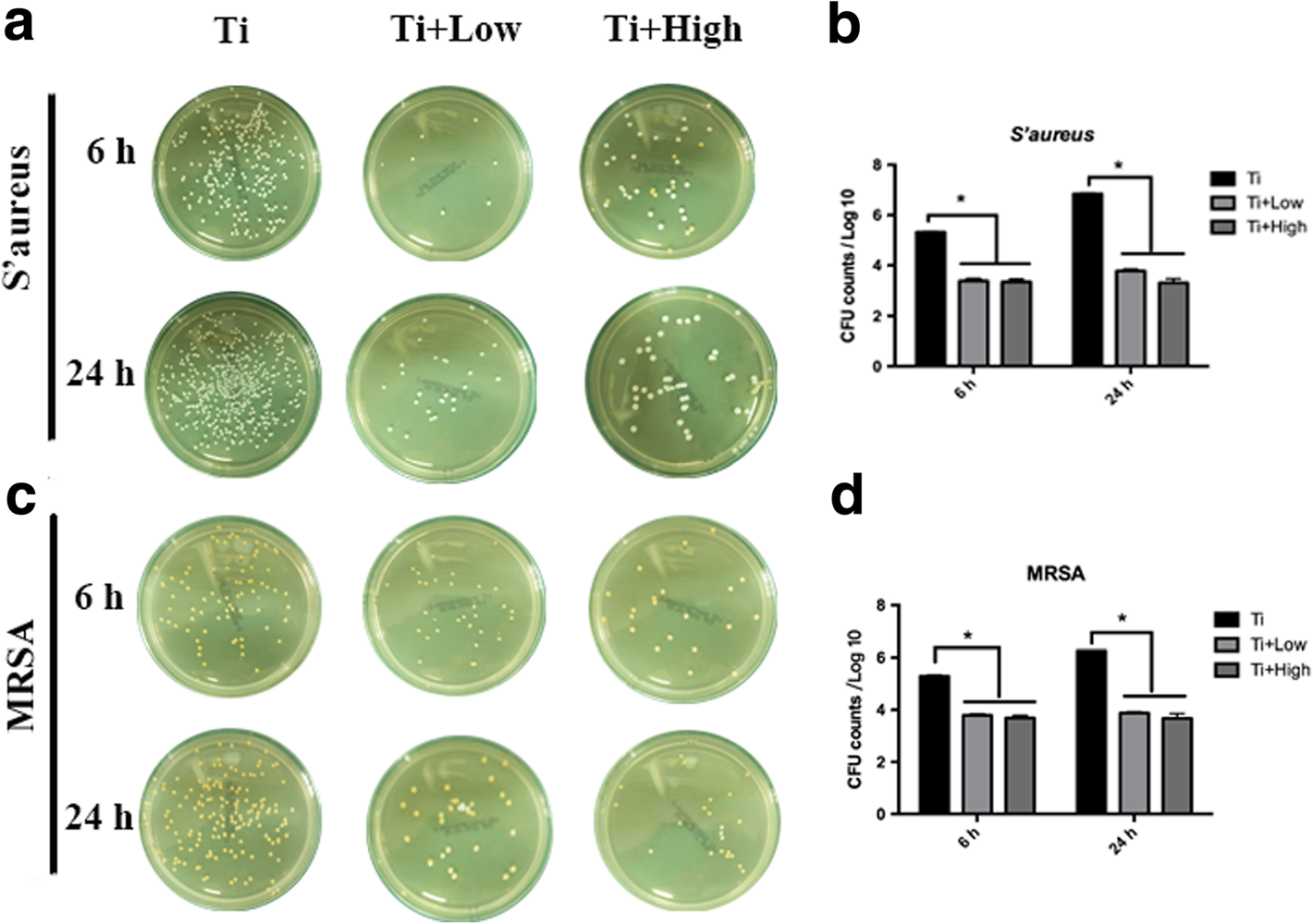
Spread-plate method to quantitatively characterize the number of viable bacteria adhered on the flat titanium with different concentration of forsythiaside at 6 and 24 h. **a** and **b**: Spread-plate method and quantitatively characterize the number of S’aureus. **c** and **d**: Spread-plate method and quantitatively characterize the number of MRSA.The asterisk denotes the significant differences in the comparison of Ti, Ti + low, and Ti + high (*p* < 0.05)

The number of viable *S. aureus* and MRSA on the Ti + low and Ti + high discs were significantly lower than those on the control Ti surfaces at 6 and 24 h (*p* < 0.05, Fig. 1). The control Ti discs showed higher numbers of viable *S. aureus* and MRSA than Ti + low and Ti + high discs (*p* < 0.05, Fig. 1). The antibacterial activity of Ti, Ti + low, and Ti + high concentration of forsythiaside was > 90% for *S. aureus* and MRSA at each time point. Thus forsythiaside could significantly inhibit the viable bacteria on Ti surface.

### CLSM to detect the live or dead bacteria on Ti samples in the presence of forsythiaside

**Fig. 2:**
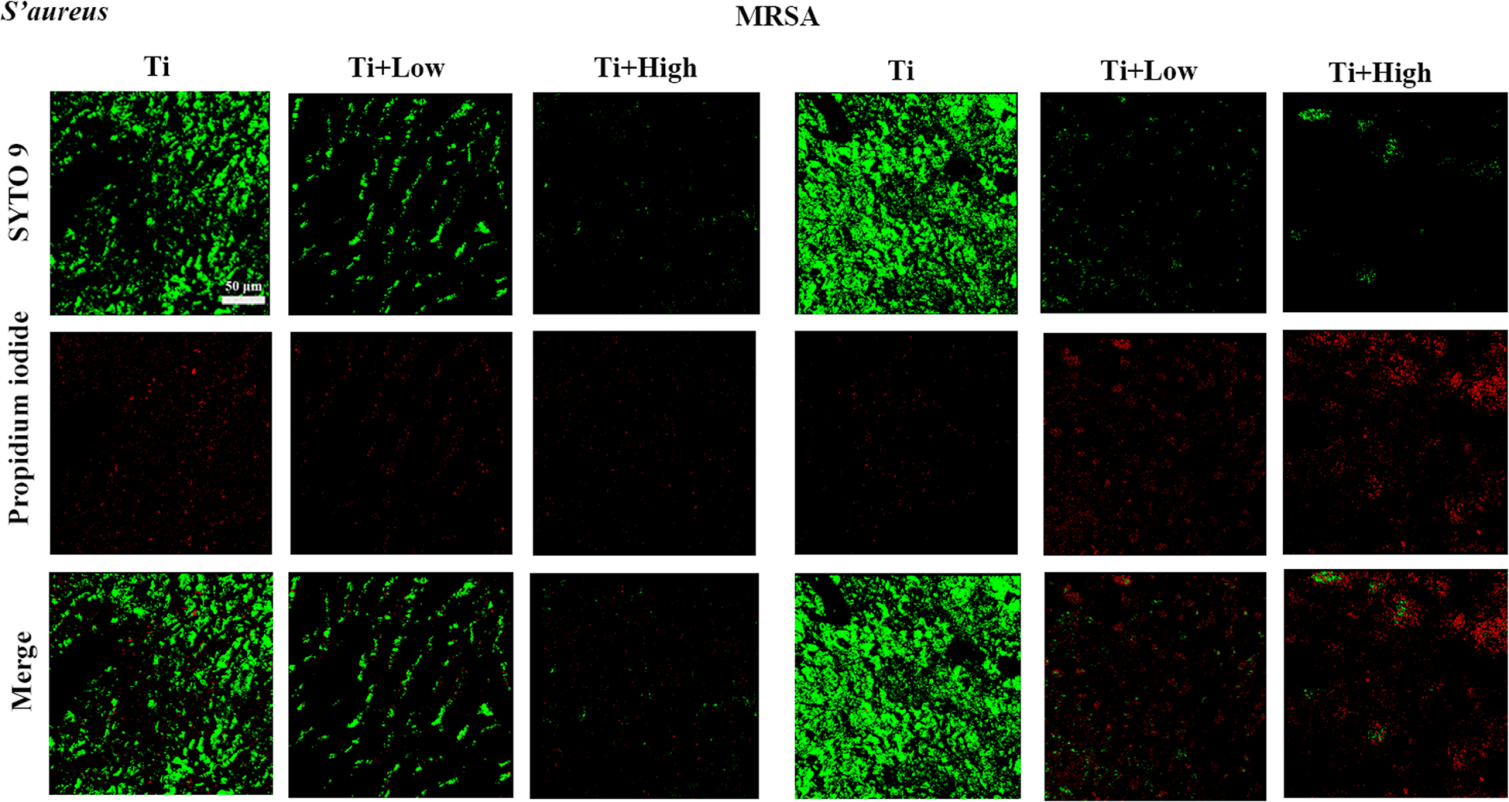
Confocal laser scanning microscopy images of bacterial (A: *S. aureus*, B: MRSA) adhesion and colonization on the surface of Ti in the presence of forsythiaside after staining with the Live/Dead Baclight™ bacteria viability kit

**Fig. 3:**
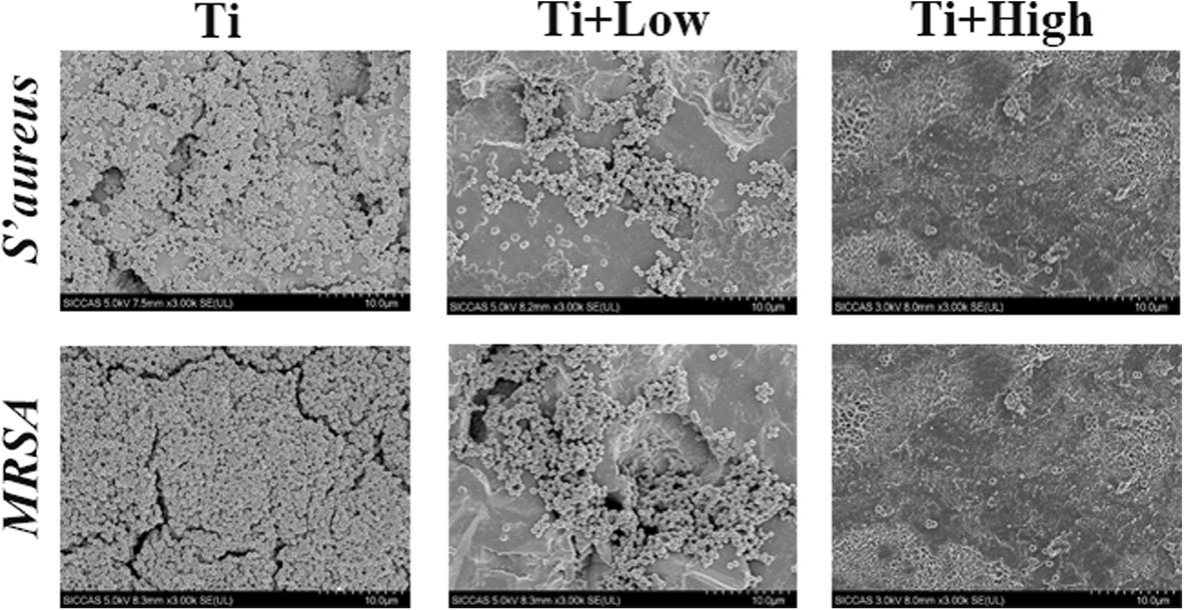
Scanning electron microscopy images of bacterial (A: *S. aureus*, B: MRSA) adhesion and colonization on the surface of Ti in the presence of forsythiaside

Live/dead cell staining assays revealed that many viable bacteria were present on the Ti surface after 24 h of co-culture of each Ti sample with *S. aureus* and MRSA in the presence of different concentrations of forsythiaside. However, the Ti + low and Ti + high groups showed significantly reduced *S. aureus* and MRSA adhesion and growth (Fig. 2). The bacteria adhering to the Ti surface at 24 h were predominantly dead, as indicated by red staining (Fig. 2), showing that forsythiaside could eliminate bacteria on the Ti surface. SEM was further used to characterize the antibacterial properties. The control Ti harbored many adherent bacteria and multiple small bacterial colonies at 24 h, whereas the Ti + low and Ti + high groups had very low numbers of adherent bacteria at 24 h (Fig. 3).

### Macrophage cell viability on the Ti surface in the presence of forsythiaside

**Fig. 4:**
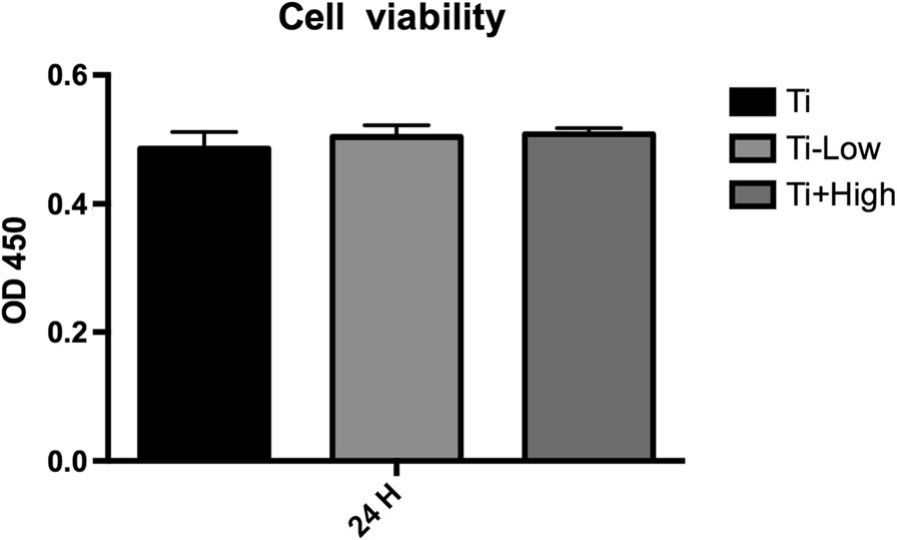
Effects of different concentrations of forsythiaside on viability of THP-1-deprived macrophages on Ti. Cell viability was measured in THP-1-deprived macrophages in Ti, Ti + low, and Ti + high groups

The cell proliferation of the samples at 24 h did not differ. No significant differences in cell attachment or proliferation were observed in the early time points between the Ti, Ti + low, and Ti + high groups in the presence of forsythiaside (Fig. 4).

### Expression of inflammatory cytokines and M1 macrophage polarization

**Fig. 5:**
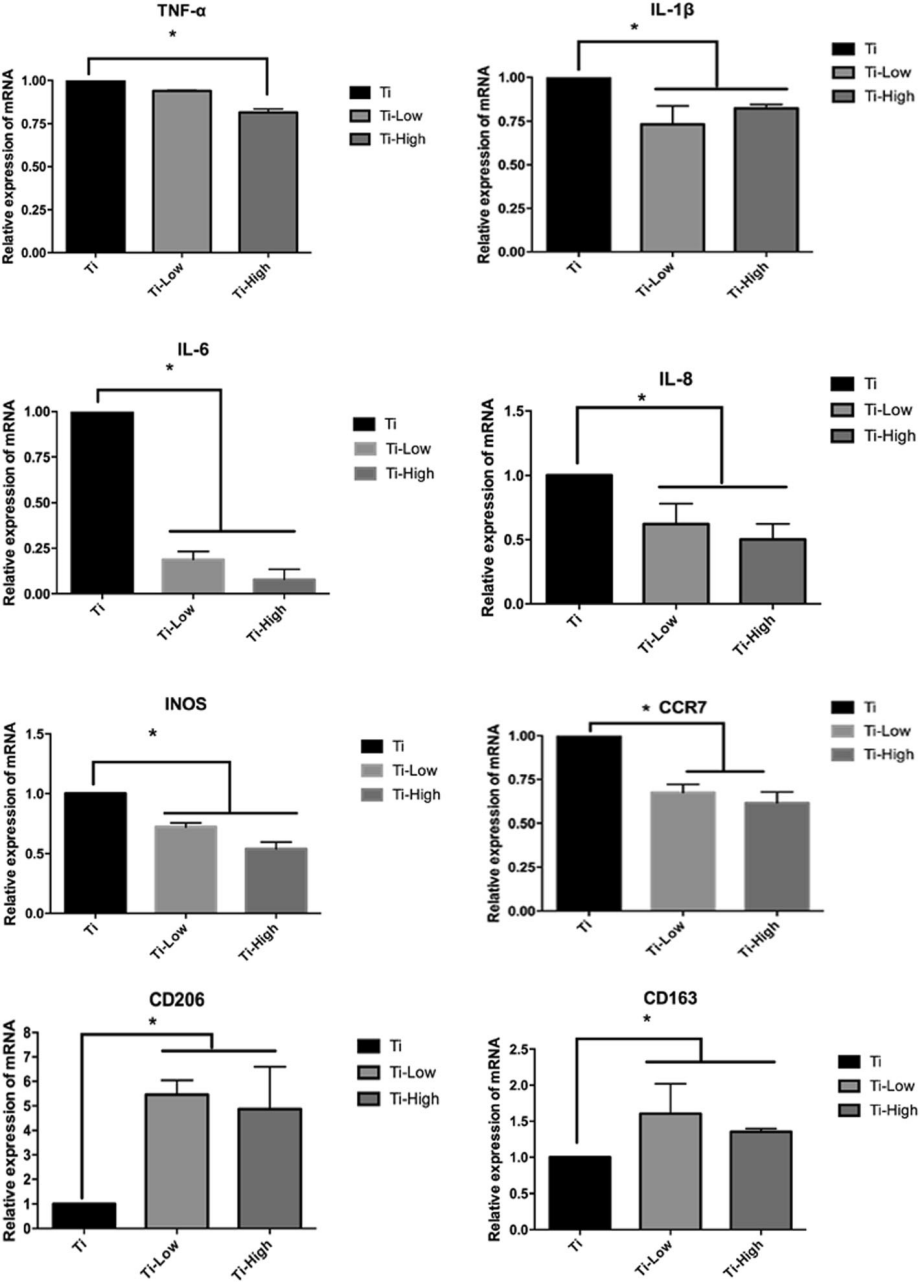
TNF-α, IL-1β, IL-6 and IL-8 mRNA levels of THP-1-deprived macrophages were used for reverse transcription real-time PCR analysis at 24 h. M1 related gene iNOS, CCR-7 and M2 related gene CD206, CD163 were investigated. The expression levels were normalized to that of GAPDH.The data are presented as the mean ± SD. *p < 0.05

We determined the mRNA levels of inflammation-related and macrophage polarization-related genes, including TNF-α, IL-1β, IL-6, IL-8, iNOS, CCR-7, CD163, and CD206 on day 3 of incubation. The expression of TNF-α, IL-1β, IL-6, IL-8, iNOS, and CCR-7 decreased in the Ti + low and Ti + high groups compared in the Ti group, while CD163 and CD206 increased in the Ti + low and Ti + high groups compared in the Ti group (Fig. 5). To assess whether changes in the expression of inflammation-related genes might be a result of forsythiaside-induced cell death, macrophage viability was determined using the CCK-8 assay after 1 and 3 days of culture. CCK-8 analysis showed no statistically significant changes during the early stages of differentiation. Therefore, we conclude that the concentrations of forsythiaside used in this study (1–100 μM) did not exert cytotoxic effects on macrophages and that the effects on inflammation caused by forsythiaside were not related to the cell death during the culture time.

### Secretion of inflammatory cytokines

**Fig. 6:**
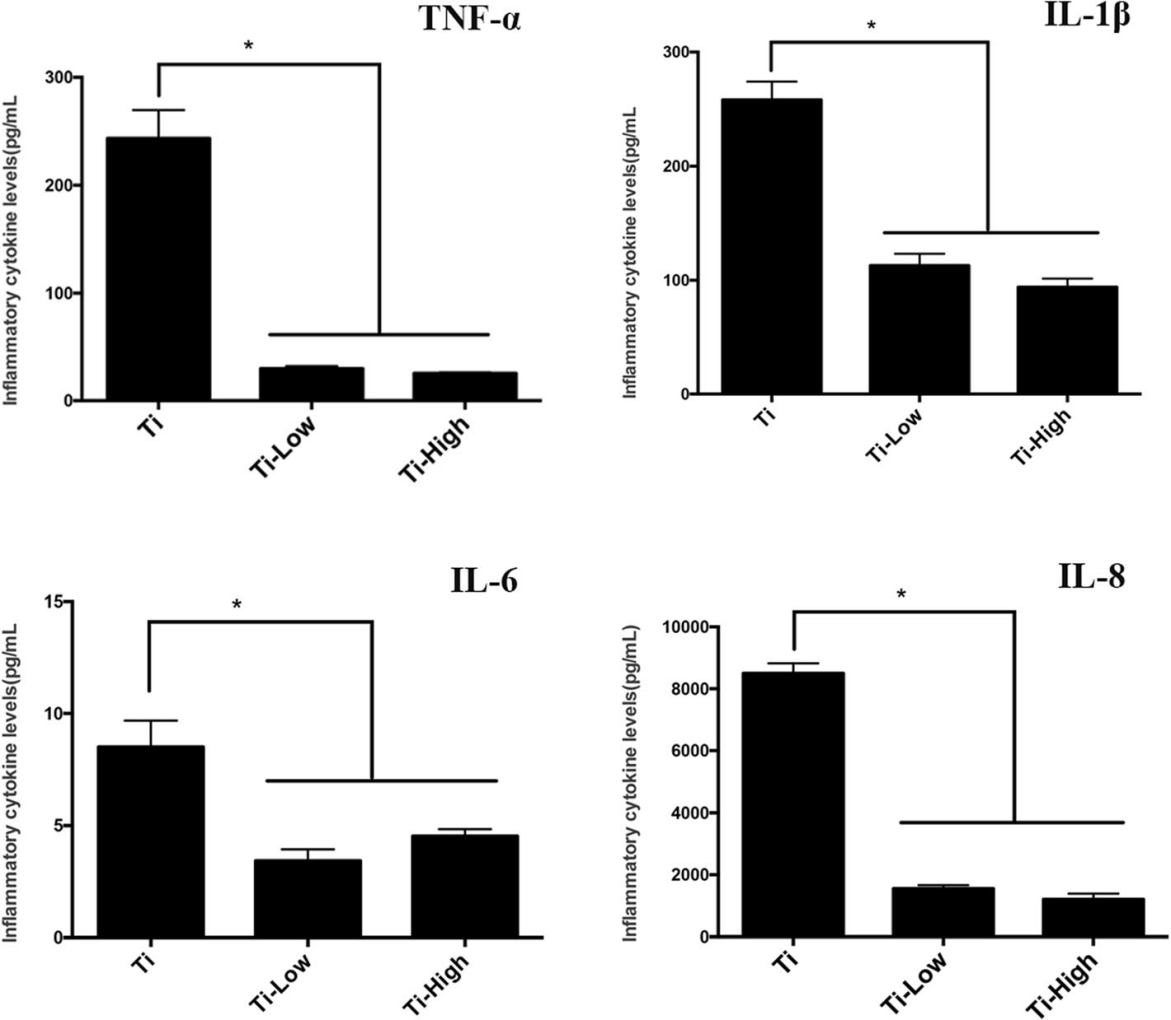
Secreted concentrations of TNF-α, IL-1β, IL-6, and IL-8 in the culture supernatant of macrophages were measured via chemiluminescence cytokines analysis at 24 h. The data are presented as the mean ± SD. *p < 0.05

We examined the inflammatory cytokine secretion on the third day of culture. The expression of inflammatory cytokines TNF-α, IL-1β, IL-6, and IL-8 decreased in the presence of low or high forsythiaside concentration (Fig. 6), demonstrating that macrophages in contact with Ti can trigger an inflammatory microenvironment that increases macrophage infiltration and release of inflammatory mediators, whereas the addition of forsythiaside could inhibit the release of inflammatory mediators.

### Macrophage morphology

**Fig. 7:**
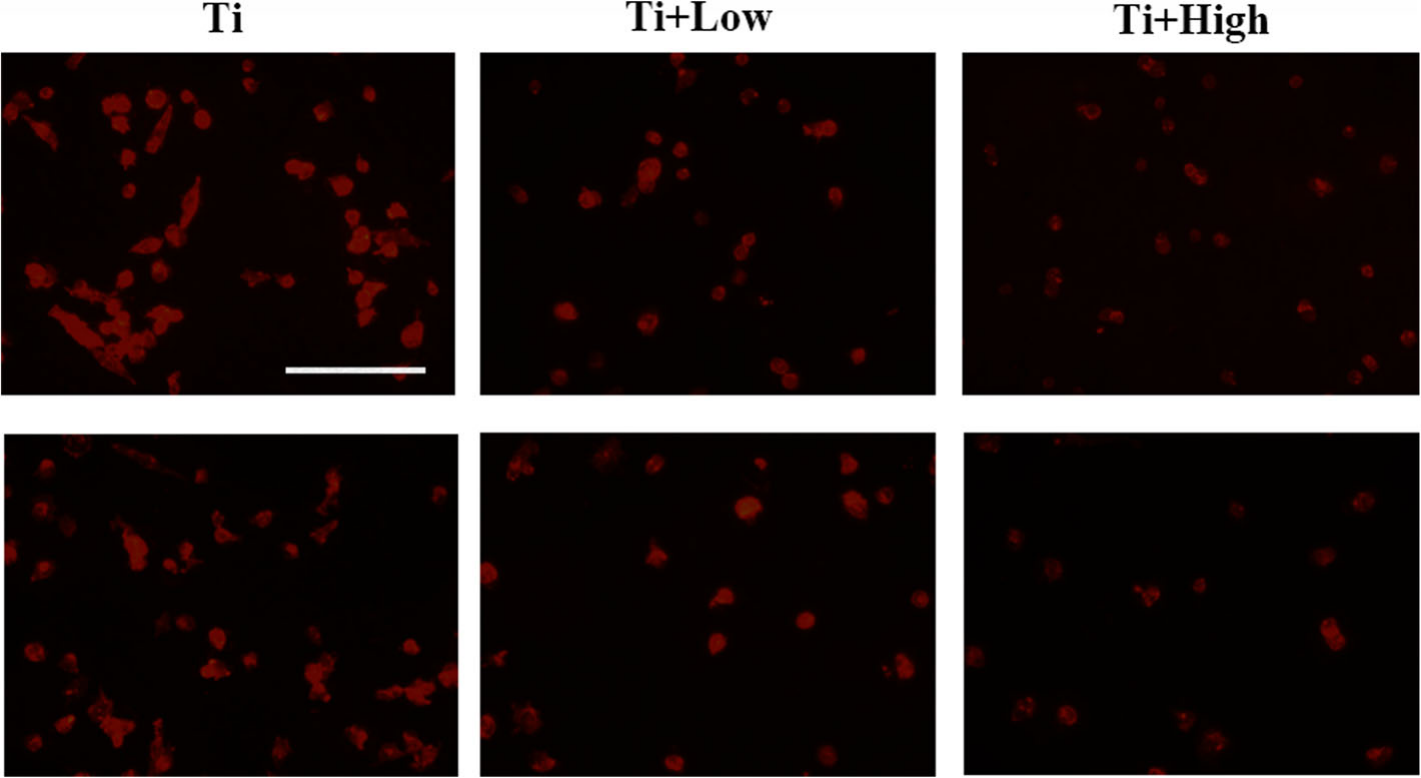
Fluorescence microscopy to evaluate the morphology of macrophages and activated macrophages on each Ti sample. The cytoskeleton is stained red. The scar bar is 50 μm

The immunofluorescence images showed that the macrophages on the blank Ti surface were in an active state, as was evident from the numerous elongated pseudopodia (Fig. 7). In contrast, the macrophages on the Ti surfaces that were pre-treated with low or high concentration of forsythiaside retained their overall native round shapes.

### NF-κB signaling pathway

**Fig. 8:**
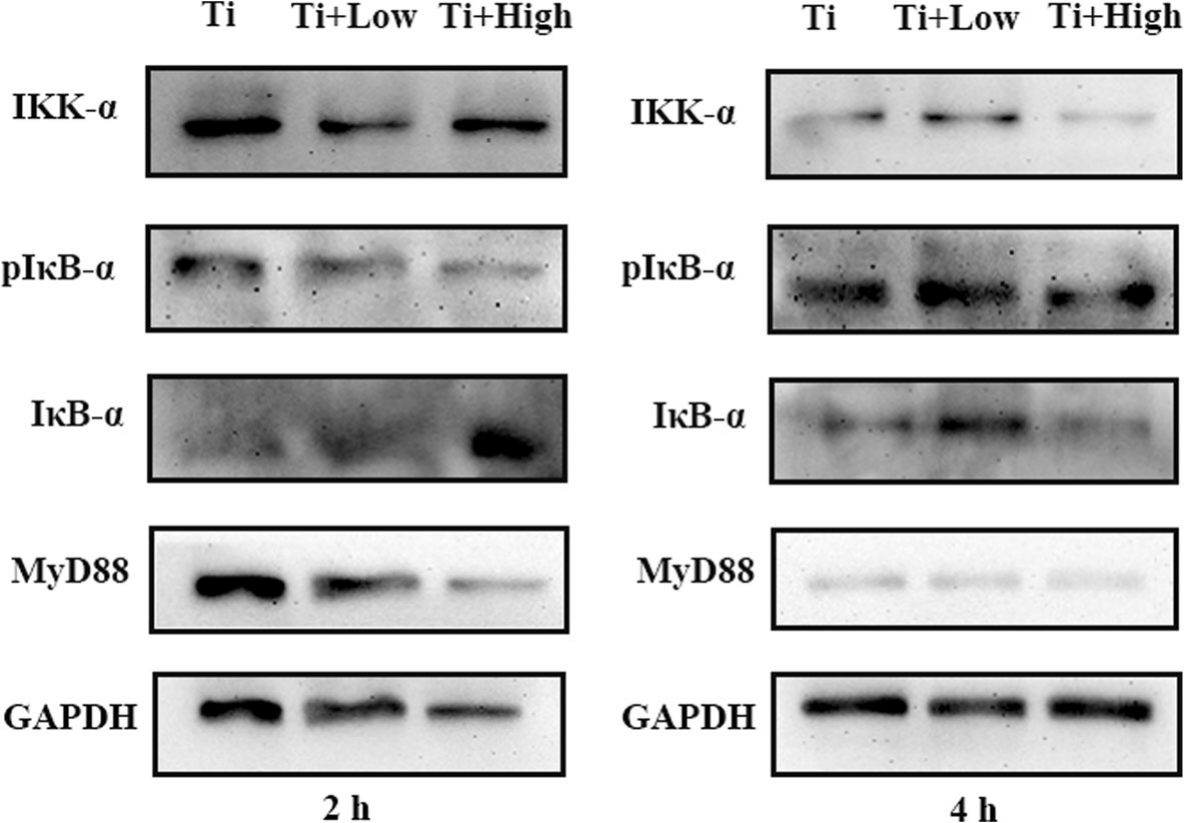
Western blot assays were conducted to detect the molecular mechanism by which FA inhibit Ti-associated inflammation at 2 and 4 h. Immunoblots displaying the phosphorylation of IκB-α in THP-1 after treatment with a series of concentration of FA for 2 and 4 h under the co-culture of Ti

Toll-like receptor 4 (TLR-4), a key pathogen recognition receptor in the innate immune system, plays an important role in the progression of inflammation. TLR-4 activation triggers an intracellular signaling pathway leading to NF-κB and inflammatory cytokine production, which is responsible for activating the innate immune system. The activation of NF-κB signaling pathway occurs primarily via activation of a kinase called the IκB kinase (IKK), which is composed of a heterodimer of the catalytic IKKα and IKKβ subunits, then increase the levels of p-IκBα and decrease the production of the inhibitor of NF-κB (IκB). With the degradation of IκB, the NF-κB complex is then freed to enter the nucleus where it can turn on the inflammation-related gene. Forsythiaside administration decreased NF-κB activation in a dose-dependent manner (Fig. 8). Moreover, treatment with forsythiaside affected the MyD88 proteins levels, whereas it decreased the levels of p-IκBα which increased the production of the inhibitor of NF-κB (IκBα) (Fig. 8). These observations suggested that forsythiaside probably targeted IKK-α and IKK-β kinases, thereby inhibiting downstream activation of NF-κB and secretion of inflammatory cytokines.

## Discussion

Improvements and innovation in medical implant material have considerably aided orthopedic diagnosis and boosted treatment technology. However, these innovations act as “double-edged swords,” as their widespread application is accompanied by new medical problems, such as implant infection. Reports show that the incidence of surgical infections of orthopedic implants is approximately 5%, of which the rate of postoperative closure infection is 3.6–8.1%, whereas that of the open fractures is as high as 17.5–21.2% [12]. Infection may occur locally after an orthopedic implantation surgery, which might affect wound healing. In addition, it can also lead to destruction of bone structure and loss of bone mass, eventually resulting in loosening of the implant.

Infection and implant-associated inflammation are the main complications that arise after an orthopedic implantation. Once the implant contacts the tissue, it is at risk of bacterial contamination, which may result in implant failure. Further, the host immune system may trigger an inflammatory response post-implantation [13, 14]. The innate immune response to the implant and secretion of inflammatory cytokines play a crucial role in determining the in vivo performance of the implant. TNF-α, IL-1β, and IL-6 are pro-inflammatory cytokines that promote apoptosis of osteoblasts, inhibit osteoblast differentiation and the expression of osteoblast differentiation-related genes, and inhibit osteogenesis of mesenchymal stem cells. Studies have reported that TNF-α and IL-1β inhibit the osteogenesis of MSCs and suppress osteoblast-related gene expression. IL-1β stimulates bone resorption by promoting osteoclast activation and mediates the osteoclastogenic effects of TNF-α by enhancing the expression of RANKL [15–17]. IL-6 can promote osteoclast differentiation and inhibit osteoblast differentiation and mineralization of the ECM of osteoblasts [18]. In addition, IL-8, an inflammatory cytokine that is mainly produced by macrophages, neutrophils, and endothelial cells, shows the potential to activate osteoclastic differentiation and bone resorption [19–21]. Therefore, it is critical to reduce the secretions of such inflammatory cytokines when macrophages come in contact with the implant. Inflammatory response-induced bone destruction occurs during orthopedic infection. In addition, bone destruction by local bacterial metabolism is a pathological process accompanying bone and joint infections, which cannot be ignored. Persistent infection and inflammatory response can activate a large number of osteoclasts, resulting in bone degradation and absorption [22–24].

Forsythiaside, an active constituent isolated from the Chinese medicinal herb *Forsythia suspensa*, exhibits anti-infective and anti-inflammatory effects. Our study demonstrated that forsythiaside inhibited *S. aureus* adhesion on Ti surface. Therefore, forsythiaside can be used locally to suppress *S. aureus* adherence to the prosthetic surface. In addition, the expression of inflammatory cytokines TNF-α, IL-1β, IL-6, and IL-8 decreased in the presence of low or high forsythiaside concentration. In view of the postoperative bacterial infection of osteoblasts and the osteoclast-mediated activation of inflammatory environment [25], the dual efficacy of forsythiaside (broad-spectrum antibacterial activity and inflammation inhibition) makes it an ideal candidate for postoperative infection control. We observed that dose-dependent forsythiaside treatment attenuated IL-1β, IL-6, TNF-α, and IL-8 expression via NF-κB suppression. TLR-4 activation upon macrophages contact with Ti. Subsequently, the activated kinase called the IκB kinase (IKK), which is composed of a heterodimer of the catalytic IKKα subunits, increased the levels of p-IκBα and decreased the production of the inhibitor of NF-κB (IκB) [26]. Forsythiaside was capable of attenuating Ti-induced activation of NF-κB signaling, targeting IκB kinase-α (IKKα) kinases of macrophages, and influencing the expression of NF-κB downstream cytokines. Therefore, forsythiaside may be used as a potential agent for the treatment of Ti implant-associated infection and related inflammation.

## Conclusions

Direct colony counting showed that forsythiaside significantly inhibited *S. epidermidis* adhesion on the Ti alloy discs in 2 h in the presence of 16 μg/mL forsythiaside. CLSM and SEM showed that forsythiaside could effectively prevent the formation of *S. aureus* and MRSA biofilm on the surface of the titanium disc in 24 h. Further, forsythiaside was capable of attenuating Ti-induced activation of NF-κB signaling, targeting IκB kinase-α (IKKα) of macrophages, and influencing the expression of NF-κB downstream inflammation cytokines. Thus, our study demonstrates that forsythiaside may be used as a potential agent for the treatment of Ti implant-associated infection and inflammation.

## Abbreviations

CFU: Colony-forming units
CLSM: Confocal laser scanning microscopy
FA: Forsythiaside
FBS: Fetal bovine serum
IKKα: IκB kinase-α/β
MHB: Mueller–Hinton broth
MIC: Minimum inhibitory concentration
MRSA: Methicillin-resistant *Staphylococcus aureus*
NF-κB: Nuclear factor-κB
RT-PCR: Real-time polymerase chain reaction
*S. aureus*: *Staphylococcus aureus*
SEM: Scanning electron microscopy
Ti: Titanium
